# High Plasma Level of Long Pentraxin 3 (PTX3) Is Associated with Fatal Disease in Bacteremic Patients: A Prospective Cohort Study

**DOI:** 10.1371/journal.pone.0017653

**Published:** 2011-03-10

**Authors:** Reetta Huttunen, Mikko Hurme, Janne Aittoniemi, Heini Huhtala, Risto Vuento, Janne Laine, Juulia Jylhävä, Jaana Syrjänen

**Affiliations:** 1 Department of Internal Medicine, Tampere University Hospital, Tampere, Finland; 2 University of Tampere Medical School, University of Tampere, Tampere, Finland; 3 Centre for Laboratory Medicine, Pirkanmaa Hospital District, Tampere, Finland; 4 Department of Microbiology and Immunology, University of Tampere Medical School, Tampere, Finland; 5 School of Health Sciences, University of Tampere, Tampere, Finland; Institut de Pharmacologie et de Biologie Structurale, France

## Abstract

**Introduction:**

Long pentraxin 3 (PTX3) is an acute-phase protein secreted by various cells, including leukocytes and endothelial cells. Like C-reactive protein (CRP), it belongs to the pentraxin superfamily. Recent studies indicate that high levels of PTX3 may be associated with mortality in sepsis. The prognostic value of plasma PTX3 in bacteremic patients is unknown.

**Methods:**

Plasma PTX3 levels were measured in 132 patients with bacteremia caused by *Staphylococcus aureus*, *Streptococcus pneumoniae*, β-hemolytic streptococcae and *Escherichia coli,* using a commercial solid-phase enzyme-linked immunosorbent assay (ELISA). Values were measured on days 1–4 after positive blood culture, on day 13–18 and on recovery.

**Results:**

The maximum PTX3 values on days 1–4 were markedly higher in nonsurvivors compared to survivors (44.8 vs 6.4 ng/ml, p<0.001) and the AUC^ROC^ in the prediction of case fatality was 0.82 (95% CI 0.73–0.91). PTX3 at a cut-off level of 15 ng/ml showed 72% sensitivity and 81% specificity for fatal disease. High PTX3 (>15 ng/ml) was associated with hypotension (MAP <70 mmHg)(OR 7.9;95% CI 3.3–19.0) and high SOFA score (≥4)(OR 13.2; 95% CI 4.9–35.4). The CRP level (maximum value on days 1 to 4) did not predict case fatality at any cut-off level in the ROC curve (p = 0.132). High PTX3 (>15 ng/ml) remained an independent risk factor for case fatality in a logistic regression model adjusted for potential confounders.

**Conclusions:**

PTX3 proved to be a specific independent prognostic biomarker in bacteremia. PTX3 during the first days after diagnosis showed better prognostic value as compared to CRP, a widely used biomarker in clinical settings. PTX3 measurement offers a novel opportunity for the prognostic stratification of bacteremia patients.

## Introduction

Bacteremia is a common disease associated with significant mortality [1]. Early categorization of patients with different prognoses is difficult in the absence of a timely sensitive and specific biomarker. Although C-reactive protein (CRP) has been widely used as a prognostic marker in infectious diseases, its prognosic value in bacteremia and sepsis is weak [2].

Pentraxins are multi-functional pattern-recognition receptors (PRR) interacting with selected viral, fungal and bacterial components. Long pentraxin 3 (PTX3), CRP and serum amyloid P (SAP) are the key members of the pentraxin superfamily. Although the long (PTX3) and short pentraxins (CRP and SAP) share common sequences they are encoded by different genes and are differentially regulated. CRP is produced in the liver, while PTX3 is an inflammatory mediator produced by various cells in peripheral tissues, for example macrophages, dendritic cells, endothelial cells, ovarian granulosa cells, fibroblasts, adipocytes and smooth muscle cells in response to the proinflammatory signals lipopolysaccharide (LPS), interleukin-1 (IL-1) and tumor necrosis factor-alfa (TNF-alfa) and Toll-like receptor activation [3], [4]. PTX3 behaves as an acute-phase protein, as its blood levels, low in normal conditions (<2 ng/ml in humans), increase rapidly in the plasma during inflammation (sepsis, endotoxin shock and other inflammatory conditions) [3]. PTX3 released in response to microbial recognition can bind specific pathogens such as fungi, bacteria and viruses, promoting phagocytosis and subsequent clearance of the pathogen via its binding to complement component C1q to induce classical complement activation [3], [5], [6]. Thus, PTX3 has a non-redundant role in the regulation of the innate immune response by contributing to the opsonization and clearance of apoptotic or necrotic cells [3].

In recently published studies, a high PTX3 level has been shown to be associated with mortality in severe sepsis and septic shock [7], and to be an early indicator of shock in severe meningococcal disease [8]. Elevated plasma levels of PTX3 predict disease severity in dengue virus infection [9] and in leptospirosis [10]. In critically ill patients PTX3 correlates with severity of disease and infection [11]. It has been shown to predict bloodstream infection and severe disease in febrile patients admitted to emergency departments [12] and indicates acute respiratory distress syndrome (ARDS) in critically ill patients [13]. PTX3 has been shown to act as biomarker of acute lung injury [14]. However, to the best of our knowledge no study has investigated the prognostic value of PTX3 in a cohort of patients with bacteremic infection.

We have previously studied prognostic factors associated with case fatality in bacteremia and found obesity (BMI≥30) and smoking to be associated with poor outcome [15]. We sought here to assess the prognostic value of plasma PTX3 in relation to other known prognostic factors in bacteremic patients. The prognostic values of two members of the pentraxin superfamily, CRP and PTX3, were compared. We show that PTX3 measurement may offer novel opportunities for the early prognostic stratification of bacteremic patients.

## Materials and Methods

### Patients

The study material comprised 132 adult patients with bacteremia admitted to Tampere University Hospital, Tampere, Finland, from June 1999 to February 2004 (Table 1). Patients were recruited from the emergency room, intensive care unit (ICU) and medical wards of the hospital. Patient recruitment, clinical data collection and sample collection were prospective. Samples for PTX3 were analyzed after hospitalization.

**Table 1:** Baseline characteristics of the study population (132 patients).

Character	
Age, median (range)	62 (18–93 years)
Gender (female/male)	62/70
**Causative organism**	
*S. aureus*	32 (24%)
*Str. pneumoniae*	37 (28%)
B- hemolytic streptococcus	22 (17%)
*E. coli*	41 (31%)
**Focus of infection (one patient may have several focuses)**	
Lung	33 (25%)
Skin	33 (25%)
Urinary	29 (22%)
Osteomyelitis/spondylitis	13 (10%)
Other or unknown focus	41 (31%)
BMI (kg/m^2^), median (range)^a^	26 (15–39)
Diabetes mellitus (type 1 or 2)	33 (25%)
Current smoking^b^	33 (28%)
Alcohol abuse	21 (16%)
Cancer (solid or hematological)	23 (17%)
At least one chronic disease	107 (81%)
McCabe class II or III^c^	22 (17%)
Cardiac disease^d^	41 (31%)
SOFA score, median (quartiles)^e^	2 (1–6)
ICU treament^f^	42 (32%)
Died (d-30 case fatality)	18 (14%)

a BMI data available on 101 patients,

b smoking data available on 120 patients,

c rapidly or ultimately fatal disease,

d valvular, coronary artery disease, heart failure or cardiac myopathy,

e sequential organ failure assessment,

f intensive care unit.

In our hospital blood cultures are routinely taken in cases with symptoms or signs of systemic infection (fever or hypothermia, tachycardia or tachypnea combined with leukocytosis or leukopenia and/or elevated C-reactive protein (CRP)). The BACTEC 9240 (BD Diagnostic Systems, Sparks, MD, USA) blood culture system was used with standard media. Patients were identified according to microbiological blood culture finding, and only those with bacteremia caused by *S. aureus, Str. pneumoniae*, β-hemolytic streptococcus or *E. coli*, the most common causative organisms in community-acquired bacteremia, were included in the study, other microbes being excluded beforehand. Blood culture-negative patients with or without sepsis syndrome and those not consenting were not included. All patients included in the study had verified infection. Only patients at least 16 years of age were enrolled. The clinicians (J.S. or J.L.) were informed by the clinical microbiologist (R.V.) of a positive blood culture from Mondays to Thursdays and the patients were enrolled in the study whenever possible to adjust to the daily schedule. We were able to recruit zero to two patients per week during the study period. Since the clinicians had no knowledge of details regarding the patients or their disease severity prior to recruitment, selection was based solely on the blood culture finding. Upon notification by the clinical microbiologist the clinicians (J.L. and J.S.) asked patients to participate and interviewed and examined those consenting. Information was gathered from hospital records at the time of a hospital visit and hospital records were also reviewed subsequent to hospitalization (R.H.). Altogether 149 out of 152 patients agreed to participate. Samples for PTX3 determinations during 1–4 days after positive blood culture were available in 132 cases, and these patients were recruited as the final study population. The study was approved by the Ethics Committee of Tampere University Hospital. Written informed consent was obtained from patients or first-degree relatives.

### Underlying diseases and chronic conditions

Chronic diseases and sources of bacteremia were registered. Calculation of body mass index (BMI, kg/m^2^) was based on weight and height as reported by the patient on admission. Patients were defined as obese if their BMI was ≥30 kg/m^2^. Alcohol abuse was defined as consumption of 300 g absolute alcohol per week or a known social or medical problem due to alcohol use. Patients were defined as current smokers and nonsmokers, i.e. those who had never smoked or had stopped smoking. McCabe classification [16] was used to determine the severity of any underlying disease.

### Collection of clinical and laboratory data

Clinical data and laboratory findings were registered on admission and during 6 consecutive days. Alterations in mental status were evaluated on the Glasgow Coma Scale (GCS), possible mechanical ventilation and the need for intensive care unit (ICU) treatment were recorded. Mean arterial pressure (MAP) ((systolic+2 x diastolic blood pressure)/3) and SOFA score (sequential organ failure assessment) [17] were calculated. The maximum SOFA score (days 0–6) for every patient was used in analysis. Disease severity was assessed by SOFA score, severe disease being defined as a score ≥4. Laboratory tests included plasma C-reactive protein (CRP, mg/l), blood platelets (x10^9^/l), plasma bilirubin (µmol/l), plasma creatinine level (µmol/l) and blood leukocyte count (x10^9^/l). The case fatality rate was studied within 14 and 30 days after a positive blood culture (d–14 and d–30 case fatality).

### Determination of PTX3 plasma levels

EDTA plasma samples for PTX3 determination were taken during patientś hospitalization and were stored at −70° until analyzed. PTX3 concentrations were determined in EDTA-plasma using a commercial solid-phase enzyme-linked immunosorbent assay (ELISA) according to the manufacturer's instructions (Quantikine DPTX 30; R&D Systems Inc., Minneapolis, USA). Samples in which the PTX3 concentration exceeded the detection range (n = 59) were serially diluted in assay diluent until they reached the dynamic range of the assay. According to the manufacturer, the mean detection limit for PTX3 is 0.025 ng/ml and the assay exhibits no cross-reactivity with either CRP or serum amyloid P. The plates were read with a Multiskan Ascent photometer (Thermo Scientific, Waltham, MA, USA) at 450 nm and corrected for readings at 540 nm.

Samples for PTX3 determinations were taken in the acute phase (days 1 to 4) (n = 132 patients), on day 13–18 (13–18 days after blood culture) (n = 73 patients) and on recovery (>25 days after positive blood culture) (n = 89 patients). Multiple samplings in the same patient were always performed on separate days. The maximum values for PTX3 for every patient measured during 1–4 days after positive blood culture were determined. Since patient recruitment was based on blood culture, which only became positive the following day, no samples for PTX3 were available on day 0 (blood culture day).

### Statistical analysis

An SPSS package (version 7.5 and version 10) was used for statistical analyses and a two-sided p-value <0.05 was taken as cut-off for statistical significance. Categorical data were analyzed by *X^2^* test or Fisheŕs exact test when appropriate, nonparametric data by Mann-Whitney U-test or Kruskal-Wallis test. A logistic regression model was used to study the independent effect of high PTX3 activity on mortality models adjusted for potential confounders. Odds ratios (ORs) were expressed with their 95% confidence intervals (CI). The survival curve was calculated using the Kaplan-Meier method and survival differences between groups were compared using the log rank test. The accuracy of maximum PTX3 value and CRP in predicting case fatality was evaluated using ROC curves [18]. In this method, a test which is perfect has 100% sensitivity and no false-positives (1-specificity = 0) and will have an area under the curve (AUC) of 1.0, whereas a test of no diagnostic value would have an AUC of 0.5. The 95% confidence intervals were calculated.

## Results

Baseline characteristics of bacteremia patients are shown in Table 1. All subjects were treated with an empiric antibiotic regimen, and when necessary antimicrobial treatment was changed according to culture results. In all patients the causative organism proved susceptible to the first empiric antibiotic treatment selected on admission. Patients received antimicrobial therapy for a median of 17 days after blood culture.

### PTX3 values in bacteremic patients

The median plasma PTX3 value in the acute phase (maximum value 1 to 4 days after blood culture) was 7.8 ng/ml (interquartile range 3.7–17.5 ng/ml) and 3.0 ng/ml on days 13–18 after blood culture (interquartile range 1.2–6.6 ng/ml). Values decreased on recovery; the median value >25 days after blood culture was 1.1 ng/ml (interquartile range 0.5–2.0 ng/ml). Of chronic conditions, alcohol abusers had higher PTX3 values compared to patients without a history of alcohol abuse (maximum values 1 to 4 after blood culture 12.6 ng/ml compared to 7.3 ng/ml, p = 0.036, respectively). However, there was no difference between groups of patients in PTX3 levels stratified by other chronic conditions, age, sex or causative organism (data not shown).

### PTX3 and the outcome of bacteremia

Median PTX3 values were significantly higher in nonsurvivors compared to survivors on days 1 to 2 (51.2 vs 9.7 ng/ml, p = 0.008), on day 3 (34.9 vs 5.3 ng/ml, p<0.001) and on day 4 (24.6 vs 4.5 ng/ml, p<0.001) after the initial diagnosis (blood culture day) (Table 2). Maximum PTX3 values on days 1 to 4 after the initial diagnosis (blood culture day) were significantly higher in nonsurvivors compared to survivors (median values 44.8 vs 6.4 ng/ml, p<0.001).

**Table 2:** Pentraxins in patients with bacteremia^a^.

Days after diagnosis	Plasma PTX3 value (ng/ml), median (quartiles)		p-value	Plasma CRP value (mg/l), median (quartiles)		p-value
	Nonsurvivors n = 18	Survivors n = 118		Nonsurvivors n = 18	Survivors n = 118	
**Day 1–2**	51.2 (40.8–113.5)	9.7 (6.4–17.7)	0.008	280 (206–368)	234 (158–335)	0.132
**Day 3**	34.9 (12.3–63.2)	5.3 (2.6–13.1)	<0.001	193 (163–238)	144 (68–232)	0.05
**Day 4**	24.6 (11.0–63.8)	4.5 (2.7–8.9)	<0.001	129 (105–160)	102 (48–168)	0.100
**Maximum value (days 1 to 4)**	44.8 (10.7–69.4)	6.4 (3.4–13.5)	<0.001	280 (206–368)	236 (155–334)	0.132

Plasma long pentraxin 3 (PTX3) and short pentraxin C-reactive protein (CRP) values 1 to 4 days after blood culture (diagnosis) in bacteremia nonsurvivors and in survivors.

a PTX3 values available for 34 patients (5 nonsurvivors and 29 survivors) on day 1–2, 81 patients (12 nonsurvivors and 69 survivors) on day 3, 104 patients (17 nonsurvivors and 87 survivors) on day 4. CRP values available for 126 patients (18 nonsurvivors and 108 survivors) on day 1–2, 85 patients (12 nonsurvivors and 73 survivors) on day 3, 109 patients (15 nonsurvivors and 94 survivors) on day 4.

**Figure 1:**
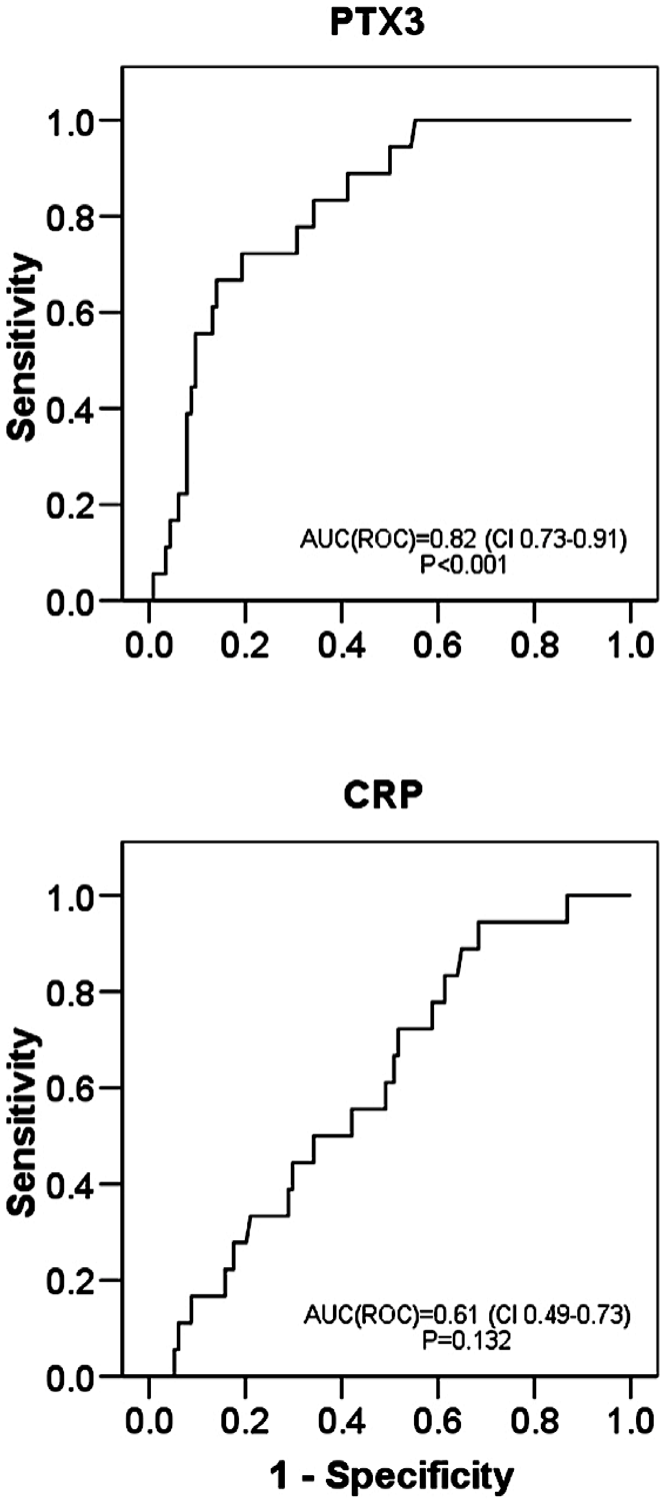
PTX3 and CRP ROC curves. Receiver operating characteristic (ROC) curves for maximal plasma long pentraxin 3 (PTX3) and C-reactive protein (CRP) levels detected on days 1–4 after positive blood culture in relation to case fatality in bacteremia patients.

**Figure 2:**
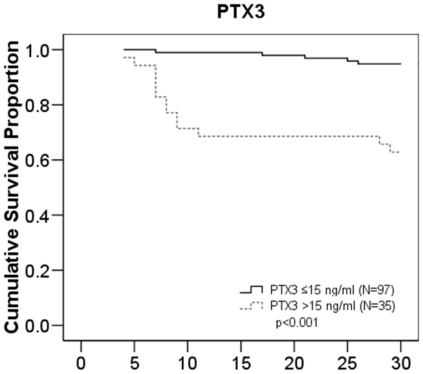
PTX3 survival curves. Cumulative 30-d survival in bacteremia patients with maximum plasma long pentraxin 3 (PTX3) level (1–4 days after blood culture) >15 ng/ml compared to those with ≤15 ng/ml. The survival curve was calculated using the Kaplan-Meier method, and survival differences between groups were compared by log-rank test.

The optimal cut-off value for the maximum PTX3 values on days 1–4 in predicting fatal disease was estimated using ROC curve, illustrated in Figure 1. The PTX3 value at a cut-off level of 15 ng/ml showed a sensitivity of 72% and a specificity of 81% in detecting fatal disease, and this cut-off point was used to classify patients into those with high or low PTX3 value. High PTX3 values were associated with several endpoints indicative of severe disease (Table 3). Figure 2 shows cumulative 30-d survival in bacteremia patients with maximum plasma long pentraxin 3 (PTX3) level (1–4 days after blood culture) >15 ng/ml compared to those with ≤15 ng/ml.

**Table 3:** Clinical characteristics of patients stratified by maximum plasma long pentraxin 3 PTX3 value (1 to 4 days after blood culture).

Characteristic	High PTX3(>15 ng/ml)N = 35	Low PTX3(≤15 ng/ml)N = 97	OR (95% CI)	p-value
Died (d-30 case fatality)	13 (37%)	5 (5%)	10.9 (3.5–33.7)	<0.001
Died (d-14 case fatality)	11 (31%)	1 (1%)	44.0 (5.4–357.7)	<0.001
Hypotensive P<70 mmHg)	26 (74%)	26 (27%)	7.9 (3.3–19.0)	<0.001
Needed ICU stay^a^	25 (71%)	17 (18%)	11.8 (4.8–29.0)	<0.001
Needed vasopressives	20 (57%)	6 (6%)	20.2 (7.0–58.6)	<0.001
Lowered Glasgow coma scale (<15)	26 (74%)	27 (28%)	7.5 (3.1–18.0)	<0.001
Needed mechanical ventilation	16 (46%)	4 (4%)	19.6 (5.9–65.1)	<0.001
Highest SOFA score≥4^b^	29 (83%)	26 (27%)	13.2 (4.9–35.4)	<0.001
Lowest MAP^c^ (mmHg), median (quartiles)	59 (52–73)	78 (68–92)	$	<0.001
Highest SOFA score, median (quartiles)	9 (4–13)	2 (0–4)	$	<0.001
Highest bilirubin level (µmol/l), median (quartiles)	23 (16–74)	17 (13–29)	$	0.006
Highest creatinine level (µmol/l), median (quartiles)	146 (91–219)	98 (75–174)	$	0.028
Median neutrophil count (x10^9^/l) (quartiles) (n = 112)	9.3 (8.1–13.2)	6.7 (3.8–9.8)	$	<0.001
Lowest platelet count (x10^9^/l), median (quartiles)	86 (28–181)	171 (113–235)	$	<0.001

a intensive care unit,

b sequential organ failure assessment,

c mean arterial pressure, $continuous variable (OR and CI cannot be applied).

The independent effect of high (>15 ng/ml) maximum PTX3 value on case fatality was studied in a logistic regression model adjusted for potential confounders (Table 4). High maximun PTX3 value was studied together with one confounder at a time in a logistic regression model, as there were only 18 patients who died. The following grouping variables have previously been shown to be associated with case fatality in a univariate model in this material: obesity, smoking, alcohol abuse, and high SOFA score (≥4)[15]. High PTX3 detected on days 1–4 after blood culture retained its significance in the logistic regression model in all combinations. Obesity and high SOFA score (≥4) also remained independent factors associated with case fatality when studied together with high PTX3.

**Table 4:** The independent effect of high maximum plasma long pentraxin 3 (PTX3) value (>15 ng/ml) on days 1–4 on case fatality in a logistic regression model adjusted for potential confounders.

Variables in the logistic regression model	Odds ratio for high PTX3 (ng/ml)
**High PTX3 (>15 ng/ml) +**	
male sex and age	11.3 (3.5–36.2)
obesity (≥30 kg/m^2^)^a^	11.1 (2.3–54.1)
alcohol abuse	10.0 (3.2–31.2)
current smoking^b^	9.9. (2.7–36.4)
McCabe class II or III	10.9 (3.5–34.0)
SOFA score (≥4)^c^	4.8 (1.4–16.4)

Obesity, smoking and alcohol abuse are included in the model as they proved to be factors significantly associated with case fatality in the univariate model in this material [15].

a BMI data available on 101 patients, obesity also remained a significant factor associated with case fatality in the logistic regression model.

b Smoking data available on 120 patients.

c Sequential organ failure assessment score; also remained a significant factor associated with case fatality in the logistic regression model.

### CRP and the outcome of bacteremia

The CRP level (maximum value on days 1 to 4) did not predict case fatality at any cut-off level in the ROC curve (p = 0.132) (Figure 1). The AUC^ROC^ for CRP (days 1 to 4 after blood culture) was 0.61 (95% CI 0.49–0.73). Thus, the optimal cut-off for CRP in detecting fatal disease could not be determined.

## Discussion

The results presented here show high PTX3 values during the first days after diagnosis to be independently associated with case fatality in patients with bacteremia. High PTX3 values were associated with several variables indicative of severe disease. Compared to CRP, a member of the same pentraxin superfamily, PTX3 may act as a more specific prognostic marker in bacteremic patients.

To the best of our knowledge, no previous study has investigated PTX3 in patients with blood culture-proven bacteremic infection. The present findings evidence the value of PTX3 in patients with blood culture-proven bacteremia caused by the four microbial organisms most commonly encountered in clinical practice. The study involved patients with non-severe disease and those admitted to the ICU due to severe infection, which enables study of the value of PTX3 in patients with distinct outcomes. In accord with the present findings, a high PTX3 level has previously been shown to be associated with mortality in severe sepsis and septic shock [7] and to be an early indicator of shock in severe meningococcal disease [8]. Furthermore, PTX3 is elevated in critically ill patients and in febrile patients admitted to the emergency room, correlating with severity of disease and infection in these patient groups [11], [12]. The present results thus confirm those of previous studies on the prognostic value of PTX3 in infectious diseases. The study shows that PTX3 may be used to predict all variables indicative of severe disease, i.e. hypotension, mechanical ventilation, low platelet count, high SOFA score, need of ICU treatment and renal failure.

PTX3 behaves as an acute-phase response protein, its blood levels being low in normal conditions (<2 ng/ml in humans) but increasing rapidly in inflammatory and infectious conditions [3]. In accord with this, the PTX3 values in the present study were high in the acute phase and decreased on recovery. Only the day 1–4 values were included in the final ROC analysis, as the PTX3 values on the first days following blood culture (i.e. diagnosis) are most unlikely to be related to conditions other than bacteremia *per se*. The initially high values already 1 to 2 days after clinical suspicion of bacteremia (after blood culture) may reflect the central role of PTX3 in the first-step innate immune response, acting as a pattern recognition receptor, with subsequent activation of complement cascade and pathogen opsonization.

In the present material, the values did not differ significantly between patients with infection caused by the four different culprit organisms. The precise clinical implications of PTX3 in infectious diseases remain elusive. In animal models, PTX3 even been found to protect from endotoxic shock and sepsis, but controversial results have also been published, highlighting the delicate balance among the various mediators which control the inflammatory response [5], [19], [20]. There is evidence suggesting that PTX3 may contribute to acute lung injury (ALI) during inflammation, and a correlation between PTX3 expression and the severity of the lung injury has been documented [14]. On the other hand, previous studies have shown that PTX3 is able to up-regulate tissue factor, a critical factor in the pathogenesis of coagulation/fibrinolysis dysregulation in sepsis [7], [8]. Sprong and associates have shown that PTX3 levels correlate significantly negatively with fibrinogen levels in sepsis. PTX3 may thus contribute to the pathological coagulation process in this condition [8]. High PTX3 concentration in severe disease may also reflect the role of pentraxins in the clearance of apoptotic cells.

The currently available biomarkers or nonspecific physiologic criteria for the sepsis syndrome or the systemic inflammatory response syndrome (SIRS) do not adequately identify patients who might benefit either from conventional antimicrobial therapies or from therapies targeting specific mediators of inflammation, i.e. recombinant human activated protein C (rhAPC) [21]. Prognosis of patients is important in risk stratification and for efficient use of hospital resources [21], [22]. The prognostic value of PTX3 as evaluated by ROC curve was better than that for CRP during the first days after diagnosis of bacteremic infection. This finding is in accord with those of a recent study in patients with severe sepsis and septic shock, showing the prognostic superiority of PTX3 over CRP [7]. Although both belong to the same pentraxin family, long pentraxins (i.e. PTX3) differ from short (SAP and CRP) in several respects, including their gene organization, chromosomal localization, cellular source and ligand-recognition and stimuli-inducing ability [5].

Some limitations must be conceded here. PTX3 values for day 0 (blood culture day) were not available. Previous study in meningococcal disease has suggested that PTX3 may already peak during the first hours after hospital admission, this reflecting disease severity, which may suggest its utility as an early marker [8]. Also *in vivo* studies indicate rapid PTX3 induction following inflammatory stimulus [5]. However, in the present study several PTX3 measurements per patient were available (median 2 measurements/patient during days 1 to 4), which reduces the possibility of bias compared to single measurement protocols. The present work was not designed to study the effects of antimicrobial therapy on PTX3 levels. Further research should also assess PTX3 levels in different settings, i.e. in patients with SIRS, in trauma patients and in viral infections. The prognostic value of PTX3 should also in subsequent studies be compared to that of procalcitonin. The biological role of PTX3 in bacteremia and sepsis calls for further elucidation. Possible interactions with coagulation process and PTX3 also warrant subsequent studies.

### Conclusions

In conclusion, PTX3 proved to be a sensitive and specific independent prognostic marker in patients with bacteremia. It may serve as a more specific indicator of severe disease than the other member of the pentraxin superfamily, CRP. PTX3 measurement may offer novel opportunities for the early prognostic stratification of bacteremia patients in order to target various therapeutic interventions.
